# Exploiting Milling By-Products in Bread-Making: The Case of Sprouted Wheat

**DOI:** 10.3390/foods9030260

**Published:** 2020-03-01

**Authors:** Gaetano Cardone, Paolo D’Incecco, Maria Cristina Casiraghi, Alessandra Marti

**Affiliations:** Department of Food, Environmental and Nutritional Sciences (DeFENS), Università degli Studi di Milano, 20133 Milan, Italy; gaetano.cardone@unimi.it (G.C.); paolo.dincecco@unimi.it (P.D.); maria.casiraghi@unimi.it (M.C.C.)

**Keywords:** bran, cell walls, sprouting, dough rheology, bread-making, microstructure

## Abstract

This research investigated the effect of sprouting on wheat bran. Bran from un-sprouted (BUW) and sprouted (BSW) wheat were characterized in terms of chemical composition, enzymatic activities, and hydration properties. In addition, the rheological properties (using GlutoPeak, Farinograph, Extensograph, and Rheofermentometer tests) and bread-making performance (color, texture, volume of bread) of wheat doughs enriched in bran at 20% replacement level were assessed. Sprouting process caused a significant decrease in phytic acid (~20%), insoluble dietary fiber (~11%), and water holding capacity (~8%), whereas simple sugars (~133%) and enzymatic activities significantly increased after processing. As regards the gluten aggregation kinetics, the BSW-blend profile was more similar to wheat than BUW-blend, indicating changes in the fiber and gluten interactions. BSW led to a worsening of the mixing and leavening properties, instead, no significant changes in extensibility were observed. Finally, BSW improved bread volume (~10%) and crumb softness (~52%). Exploiting bran from sprouted wheat might be useful to produce bread rich in fiber with enhanced characteristics.

## 1. Introduction

Fiber-enrichment of food products has become increasingly important as a means to increase their nutritional properties. In this context, bran from cereals-with a total dietary fiber content of 30%–50%—is one of the most important source of dietary fiber used in the bread-making industry [1]. However, the inclusion of high levels of fiber in cereal-based products remains a technological challenge, due to the need to maintain acceptable dough rheological properties as well as sensory attributes. Indeed, adding high levels of bran to dough leads to an increase in water absorption, a decrease in both mixing stability and leavening tolerance [2,3]. The most evident effects on the final baked product are the decrease in loaf volume, the increase in crumb firmness, the appearance of dark crumb, and, in some cases, the modification of taste with the appearance of bitterness [4]. 

The detrimental effect of bran addition on bread-making cannot be solely attributed to the dilution of gluten proteins and to the physical disruption of gluten network, but the physical, chemical, and biochemical properties of bran should be also considered [5]. Besides specific physical properties—i.e., the strong tendency of bran to absorb water that might result in competition for water between bran and other key flour components like starch and proteins—bran seems to have a certain chemical reactivity (i.e., between ferulic acid and proteins) which might determine its functionality [5].

Several pre-treatments have been proposed to counter these negative effects, such as: (i) particle size reduction, which significantly influences the rheological properties of dough in terms of mixing time, stability and dough resistance to extension [6], (ii) application of high-pressure [7], and (iii) enzymatic treatment [8], which alters the physical and structural properties of dough and its interaction with water and (iv) fermentation, which improves the bioactivity and baking properties of dough enriched with wheat bran [9]. Considering all these treatments, the best results were obtained when exogenous enzymes were used as such [6] or produced by microorganisms [9]. 

In this context, sprouting (or germination) can be proposed as a bio-technological process able to promote the accumulation of enzymatic activities. A recent study reported that wholegrain flour from sprouted wheat could be used to produce bread with improved characteristics (i.e., specific volume and crumb softness) compared to conventional wholegrain flours [10,11]. Enhancements in bread attributes were also found by using refined flour from sprouted wheat [12,13]. Specifically, Marti et al. [12] proposed the use of flour from sprouted wheat as alternative to the conventional enzymatic improvers in bread-making. Indeed, low amount of sprouted wheat flour (<2%) enhanced the bread-making attitude of stiff flour, with the advantage of producing a clean label product. 

Considering the potential use of flour from sprouted wheat, it remains to elucidate the physical, chemical, and structural aspects of the wheat milling by-products (i.e., bran) and how these characteristics might affect the baking performance of bran-enriched wheat bread. Indeed, although in the literature there is a large amount of study available on the enrichment of baked products in bran, no information is available on the use of bran from sprouted wheat in bread-making. Starting from this point, the purpose of this research was to investigate the features of bran obtained from sprouted wheat and how this ingredient affects both dough rheological properties and bread characteristics.

## 2. Materials and Methods 

### 2.1. Materials

Wheat kernels (*Triticum aestivum* L.) were kindly supplied by Molino Quaglia (Molino Quaglia S.p.A., Vighizzolo d’Este, Italy). A part of kernels (10 tons) was sprouted at industrial scale for 48 h, and then dried at 60 °C for 12 h, as previously reported by Marti et al. [13]. Both samples, un-sprouted and sprouted kernels, were milled using a laboratory mill (Labormill, BONA, Monza, Italy) to collect bran (bran yield: 20%). 

Wheat brans (bran from un-sprouted and sprouted wheat—BUW and BSW—respectively) were toasted (Self-Cooking Center^®^—Rational AG, Landsberg am Lech, Germany) at 200 °C for 120 s, in order to inactivate parts of the enzymes. Bran fractions with particle size >500 µm were further ground using the Cyclotec 1093 (FOSS, Höganäs, Sweden) to decrease their size (<500 µm). BUW and BSW were used in replacement of the 20% of a commercial refined wheat flour (CTRL; W = 280 × 10^−4^ J; P/L = 1.16) provided by Molino Quaglia (Molino Quaglia S.p.A., Vighizzolo d’Este, Italy). In this way, two whole-wheat flours were obtained which differences were associated solely to bran type (BUW or BSW).

### 2.2. Analytical Methods

#### 2.2.1. Microstructural Evaluation

Kernels from either un-sprouted or sprouted wheat were prepared for confocal laser scanning microscopy (CLSM) and light microscopy (LM) to specifically evaluate changes due to sprouting process itself. Specimens were fixed and dehydrated according to Faltermaier et al. [14] then embedded in a methacrylate resin (Technovit 7100, Wertheim, Germany). After resin polymerization, 10 µm-thick sections were obtained using a rotary microtome (Leitz 1512). Sections were treated, with 2,4-dinitrophenylhydrazine (20 min) followed by washing in tap water (30 min), then in 0.5% periodic acid (20 min) and again in tap water (30 min) [14]. This procedure is a modification of the periodic acid–Schiff’s reaction that allows the dye acid fuchsin to be specific for protein without binding to other polysaccharides like starch. 

The staining for protein and cell walls was performed by using 0.1% (*w*/*v*) water solution of acid fuchsin (Sigma-Aldrich, St Louis, MO, USA) for 1 min and 10% (*w*/*v*) Calcofluor white for 1 min (fluorescent brightener 28, Sigma-Aldrich, Milan, Italy), respectively. Sections were inspected using an inverted CLSM (Nikon A1+, Minato, Japan). Acid fuchsin was excited at 560 nm and the emission filter was set at 630–670 nm while Calcofluor white was excited at 409 nm and the emission filter was set at 430–480 nm. Starch was stained by using Lugolʼs solution (5 g I_2_ and 10 g KI in 100 mL MilliQ water), and sections were examined after 5 min staining with an Olympus BX light microscope (Tokyo, Japan) equipped with QImaging Retiga camera (Surrey, Canada).

BUW and BSW were prepared for CLSM as mentioned above and observed exploiting the auto-fluorescence of the samples. Specimens for SEM observations were prepared according to Cardone et al. [10] observed using a Zeiss LEO 1430 SEM at 3 kV.

#### 2.2.2. Chemical Composition 

Moisture content of bran was evaluated at 130 °C until the sample weight did not change by 1 mg for 60 s in a moisture analyzer (Radwag—Wagi Elektroniczne, Chorzòw, Poland). Total starch content was evaluated by standard method (AACC 76-13.01) [15]. Sugars were assessed by HPLC by Anion Exchange Chromatography with Pulsed Amperometric Detection (HPAEC-PAD) [16]. Total (TDF), soluble (SDF), and insoluble (IDF) dietary fiber contents were quantified by an enzymatic-gravimetric procedure as reported by standard method (AOAC 991.43) [17]. 

Total and soluble arabynoxylans were determined as reported by Manini et al. [18]. The phytic acid content was determined by HPLC with spectrophotometric detection as previously reported by Oberleas and Harland [19]. 

#### 2.2.3. Enzymatic Activities 

α-amylase activity of bran was evaluated by following standard method (AACC 22-02.01) [15]. The analysis of xylanase activity was performed by using the Azo-wheat arabinoxylan kit (K-AZOWAX 09/04; Megazyme International Ireland Ltd., Wicklow, Ireland) with some modifications (i.e., 1 h of incubation instead of 20 min). 

#### 2.2.4. Hydration Properties 

The water holding capacity (WHC) of bran was determined as reported by Lebesi and Tzia [20] by suspending 0.5 g of each bran samples with 45 mL distilled water. Instead, water binding capacity (WBC) was evaluated according to Zanoletti et al. [21]. 

#### 2.2.5. Rheological Properties 

The effects of bran on flour functionality and bread-making performances were determined on two blends prepared by substituting the 20% of commercial flour with BUW and BSW at the same level, respectively. Control flour without bran was also examined.

##### Gluten Aggregation Properties

The aggregation properties of gluten were investigated by means of the GlutoPeak (Brabender GmbH and Co. KG, Duisburg, Germany) test, according to Marti et al. [12]. The major indices considered were: (i) Maximum Torque (MT, expressed in Brabender Equivalents, BE), indicating the peak following the aggregation of the gluten proteins; (ii) Peak Maximum Time (PMT, expressed in s), indicating the time to obtain the Maximum Torque; (iii) Total Energy (expressed in GlutoPeak Equivalent, GPE) indicating the area under the curve from the beginning of the analysis up to 15 s after the Maximum Torque.

##### Mixing Properties

Mixing properties were evaluated following to standard method (ICC 115/1) [22] in the 50 g kneading bowl of the Farinograph-E^®^ (Brabender GmbH & Co. KG, Duisburg, Germany). 

##### Extensibility Properties 

The extensograph test was carried out on a 20 g dough by means of the micro-Extensograph^®^ (Brabender GmbH & Co. KG, Duisburg, Germany), at three resting times (45, 90, and 135 min). Dough samples were prepared by following to standard method (AACC 54-10.01) [15], in the 50 g kneading bowl of the Farinograph-E^®^ (Brabender GmbH & Co. KG, Duisburg, Germany). 

##### Leavening Properties

The Rheofermentometer^®^ (Chopin, Tripette, and Renaud, Villeneuve La Garenne Cedex, France) was used to analyze dough development and carbon dioxide (CO_2_) production and retention using the method described by Marti et al. [13]. 

#### 2.2.6. Baking Test

A straight-dough method was applied for the production of bread according to Cardone et al. [10]. The amount of water added to the sample and the mixing time of the dough varied for each formulation according to previously determined farinographic indices. The dough samples were split into sub-samples of 80 g each, formed into cylindrical shapes, put into square shape bread molds (height: 4 cm; length: 9 cm; depth: 6 cm) and left to leaven for 60 min in a thermostatic chamber at 30 °C (70% relative humidity). To prevent the formation of crust too quickly, the leavened dough was baked in an oven (Self Cooking Center^®^, Rational International AG, Landsberg am Lech, Germany) in two stages. Firstly, the samples were baked at 120 °C with vapor injection (90% relative humidity) for 4 min and then the oven temperature was increased to 230 °C for 11 min. The resulting loaves, two hours after baking, were analyzed or packaged in orientated polypropylene film for three days. 

#### 2.2.7. Bread Properties

##### Color 

The evaluation of the browning (100-L*) and saturation of the color intensity (redness and yellowness, a* and b*, respectively) of bread crust and crumb were assessed with a reflectance color meter (CR 210, Minolta Co., Osaka, Japan).

##### Specific Volume

The bread specific volume was performed through the ratio between volume and mass of bread and expressed in mL/g. The apparent volume was evaluated by the sesame displacement approach. 

##### Crumb Hardness 

Crumb hardness was assessed by means of a dynamometer (Z005, Zwick Roell, Ulm, Germany), equipped with a 100 N load cell as previously described by Marti et al. [23]. Bread samples were evaluated two hours after baking (day zero), and after one and three days of storage. Crumb hardness was determined as the maximum compression force at a deformation of 30%. Three central slices (15 mm thick) of one loaf from each bread-making trial were analyzed. The values of hardness resulting from each slice of bread were analyzed by applying the Avrami Equation (1):

(1) $$\theta =\frac{\left(A_{\infty }-A_{t}\right)}{\left(A_{\infty }-A_{0}\right)}=e^{-ktn}$$

where θ indicates the fraction of the total change in hardness still to occur, *A*_0_, *A*_t_, and *A*_∞_ are experimental values of hardness at times zero, *t* and infinity respectively, *k* is a rate constant and *n* (*n* = 1) is the Avrami exponent. All the parameters were obtained from the modelling process.

### 2.3. Statistics

Chemical composition, enzymatic activities, hydration properties, and gluten aggregation properties were determined in triplicate, whereas mixing and leavening properties were analyzed in duplicate. As regards the extensograph test, the determinations for each sample were made in duplicate and from each dough two subsamples were analyzed. For the bread production, two baking tests were carried out, and three loaves were prepared from each baking test. Color measurements were replicated five times. Bread specific volume, crumb porosity, and hardness were carried out on six loaves or slices. 

To determine differences between means (for BUW and BWS) a paired t-test was applied. Analysis of variance (one-way ANOVA) was performed by utilizing the Fisher’s Least Significant Difference (LSD) test. Data were elaborated by Statgraphic XV v. 15.1.02 (StatPoint Inc., Warrenton, VA, USA).

## 3. Results

### 3.1. Microstructure Evaluation of Kernels and Brans before and after Sprouting

**Figure 1:**
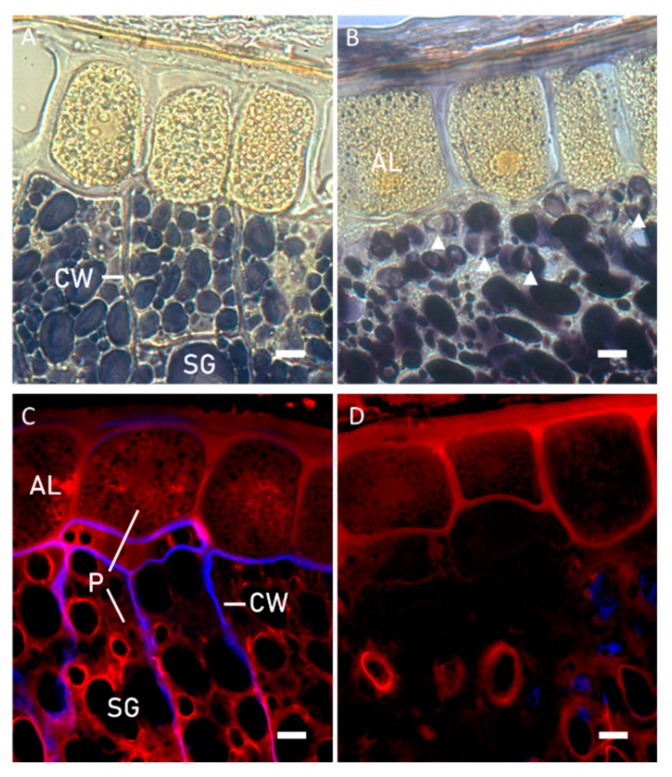
Light microscopy (**A**,**B**) and confocal laser scanning microscopy (**C**,**D**) of kernels before (**A**–**C**) and after (**B**–**D**) sprouting process. AL: aleuron layer, CW: cell wall, SG: starch granule, P: protein. White head arrows in panel B show hydrolyzed starch granules. Bars are 10 µm in length.

Microstructural modifications due to sprouting process were evaluated on the whole kernel to avoid interferences of mechanical breaks possibly brought by milling. Sprouting caused the hydrolysis of the subaleurone endosperm cell walls, that were no longer visible in sprouted kernels (Figure 1A,B), and the hydrolysis of some starch granules near the aleurone cells (head arrows in Figure 1B), consistently with the hydrolytic enzyme synthesis in the aleurone layer. Fluorescence of Calcofluor white, specific for cell walls especially β-1,4-glucans, considerably decreased in sprouted kernels (Figure 1C,D). At the same time, the decrease in fluorescence of acid fuchsin suggested a protein degradation in subaleurone region (Figure 1C,D).

**Figure 2:**
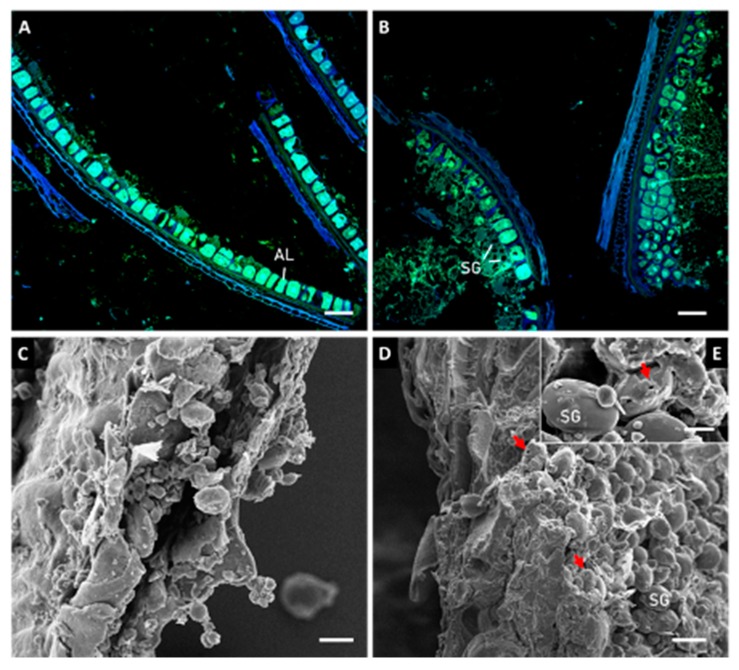
Confocal laser scanning microscopy (**A**,**B**) and scanning electron microscopy (**C–E**) of bran before (**A**–**C**) and after (**B**,**D**,**E**) sprouting process. AL: aleuron layer, SG: starch granule. Red arrows in panel D and E show the effect of amylolytic enzymes as “erosion pits” on the granule surface. Bars are 40 µm in length in panels A,B, 10 µm in length in panels C,D and 5 µm in panel E.

CLSM of brans (Figure 2A,B) clearly show that BUW is almost composed of pericarp and aleurone layers, whereas BSW is richer in starch granules since large portions of starchy endosperm remained adherent to the aleurone layer. This difference may be a consequence of different break paths after sprouting and it explains the lower flour yield of BSW (51% vs. 54%; data not shown). SEM analysis confirmed the relevant structural changes induced by sprouting and showed the hydrolysis of starch granules mainly to occur in the BSW as erosion pits (red arrow in Figure 2E). 

### 3.2. Effect of Sprouting on Bran Features 

Sprouting process increased the amount of total sugars in bran, and specifically glucose, fructose and maltose (Table 1). The higher starch content in BSW might be the consequence of the different compactness and, therefore, milling behavior of BUW and BSW, as shown in Figure 2. A significant decrease in IDF was found after sprouting (−11% in BSW respect to BUW). Instead, the SDF content of BUW and BSW was not affected by the process. Moreover, sprouting did not induce a significant degradation and solubilization of arabinoxylans. On the contrary, in the case of prolonged sprouting (i.e., four days), a significant decrease in the total content of arabinoxylans and an increase in water-extractable ones was reported in barley [24]. In contrast, the sprouting degraded antinutritive factors, such as phytic acid, confirming previous studies carried out on wholemeal flours [25].

As expected, sprouting promoted an accumulation of both α-amylases and xylanases in the bran fraction (Table 1). Specifically, the activity of α-amylase in BSW increased by about 1500-fold compared to BUW, whereas the xylanase activity in BSW was 1.2-fold higher than BUW.

BUW and BSW hydration properties were assessed in terms of their water holding (WHC) and water binding (WBC) capacity. The WHC significantly decreased after sprouting process, by about 8% (Table 1). Instead, there was no significant differences between BUW and BSW in terms of WBC. 

### 3.3. Gluten Aggregation and Mixing Properties

**Figure 3:**
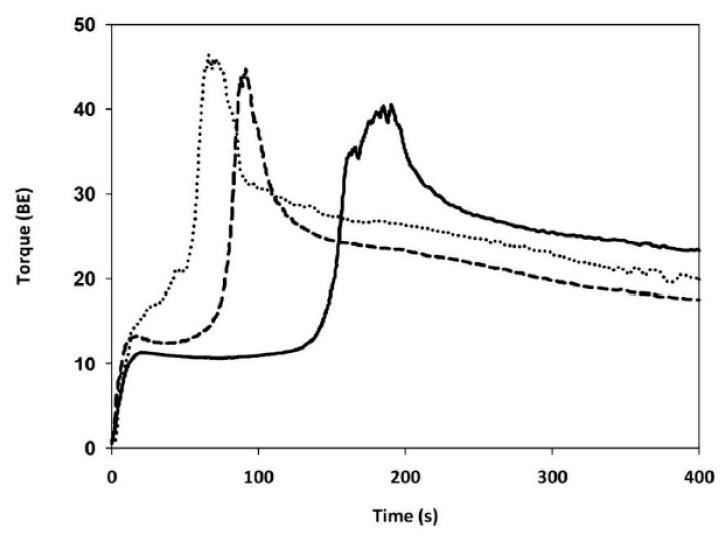
GlutoPeak profiles of refined wheat flour alone (solid line) and in presence of bran from un-sprouted wheat (dotted line) or bran from sprouted wheat (dash line).

During the GlutoPeak test, the speed of the rotating paddle allows the formation of gluten, and a rapid increase in the torque curve occurs. Additional mixing breaks down the gluten network and the torque curve declines [26]. The gluten aggregation kinetics of CTRL was typical of a strong flour with good bread-making performance that is usually characterized by long aggregation time (i.e., PMT), high torque (i.e., MT) and high energy values (Figure 3 and Table 2). 

Replacing 20% of refined wheat flour with both types of bran, the PMT and MT indices significantly decreased and increased, respectively, resulting a decrease in the energy value, suggesting gluten weakening [27]. Worsening in gluten aggregation properties where more evident when BUW was added to CTRL sample, instead of BSW.

**Figure 4:**
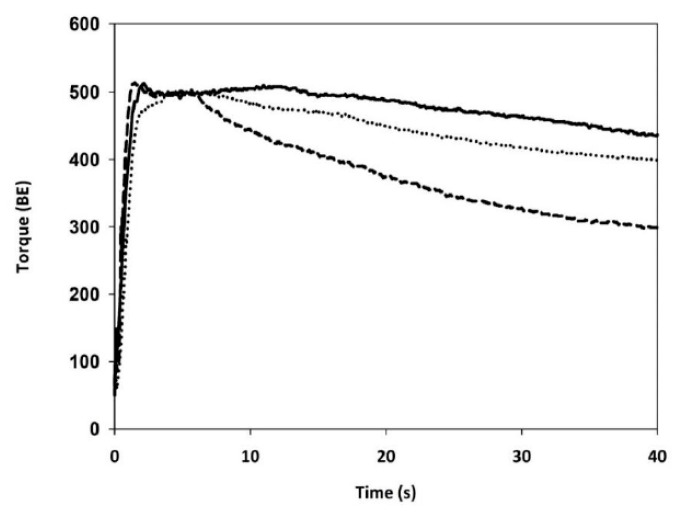
Farinograph profiles of refined wheat flour alone (solid line) and in presence of bran from un-sprouted wheat (dotted line) or bran from sprouted wheat (dash line).

The CTRL flour used for making the blends was characterized by a long dough development time and high stability (Figure 4; Table 2), in agreement with the GlutoPeak test (Figure 3 and Table 2). When a 20% of flour was replaced by BUW or BSW, a significant increase in water absorption was observed (Table 2), with BUW-blend absorbing more water than BSW-blend, in agreement with the hydration properties of the related bran samples (Table 1). Regarding the development time, the presence of BUW decreased the time needed to achieve maximum consistency (−48%). This effect was even more evident when BSW was added (−58%). Dough stability, which in wheat dough indicates dough strength, decreased significantly by about 47% and 72% in the case of BUW- and BSW-blends, respectively. In contrast to the GlutoPeak test, the farinograph test showed the highest weakening of the dough when BSW was added.

### 3.4. Dough Extensibility

**Figure 5:**
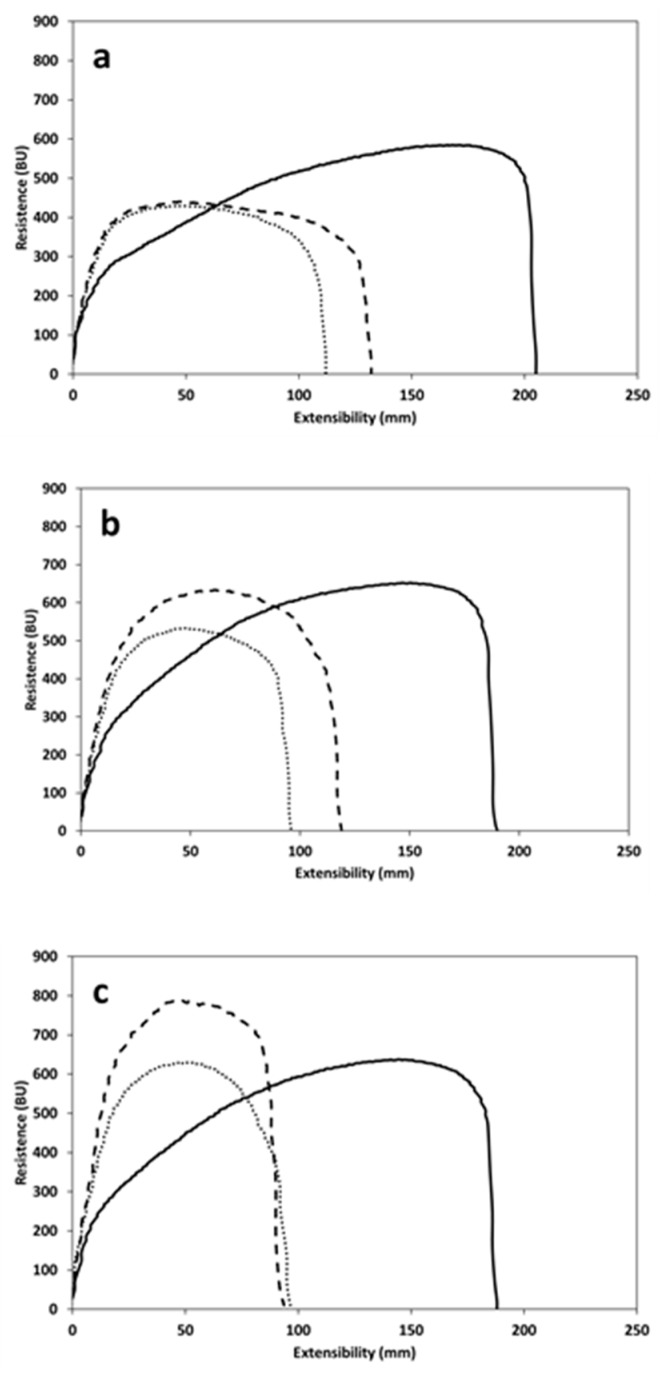
Micro-Extesograph profiles of refined wheat flour alone (solid line) and in presence of bran from un-sprouted wheat (dotted line) or bran from sprouted wheat (dash line), after 45 min (**a**), 90 min (**b**), and 135 min (**c**) of resting time.

Dough extensibility significantly decreased when bran was added, with no significant differences according to the type of bran (Figure 5a–c and Table 3). In addition, all samples showed a decrease in dough extensibility as the resting time increased, in particular when the resting time was extended from 45 min to 90 min. However, the extensibility for the CTRL flour remained practically unmodified between 90 and 135 min.

Bran-enrichment did not cause any significant modification regarding resistance at 45 and 90 min of resting time, while after 135 min of resting time, bran caused an increase in dough resistance, suggesting dough stiffness [28]. This phenomenon is more evident when BSW was included in the dough. 

The ratio number, which indicates the ratio between the resistance to extension and extensibility, increased with the addition of bran, confirming the role of this milling fraction in inducing dough stiffness. This phenomenon is well shown by the high ratio number values at 135 min. The highest increase in ratio number was recorded when BSW was added to the CTRL flour. Finally, the energy required for deformation was decreased by the addition of bran, particularly when BUW was used.

### 3.5. Leavening Properties

**Figure 6:**
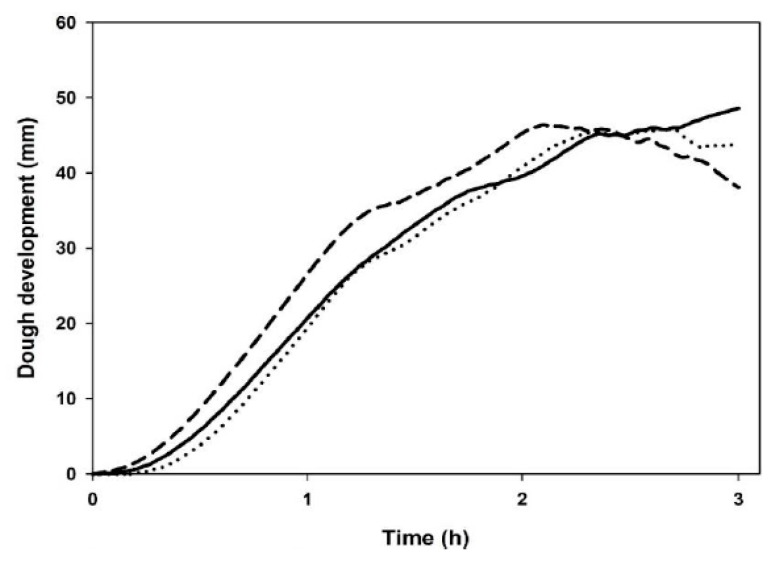
Dough development profiles of commercial wheat flour (solid line); with bran from un-sprouted wheat (dotted line) or bran from sprouted wheat (dash line).

Maximum dough height during leavening was not significantly altered by the addition of bran (Figure 6). On the other hand, the time of maximum dough development decreased, from 180 min in the control to 161 min and 131 min by adding BUW and BSW, respectively. The time of dough development and the loss in dough volume (weakening coefficient), was high for all bran-enriched samples, especially when BSW was added. The decrease in dough stability agrees with the farinograph index. The drop off after three hours of leavening ranged from 6% and 17.5% for BUW and BSW samples.

Regarding gas production and retention (Table 4), dough with bran produced more gas than CTRL and this is due to a higher content of simple sugars (Table 1), which are consumed faster by yeast compared to CTRL. This effect was more noticeable when BSW was used. As regards the gas retention coefficient (Table 4), the BUW addition did not induce a significant negative effect after three hours of leavening. In contrast, in presence of BSW the gas retention capacity decreased from 94% (CTRL) to 86%, after the same time (i.e., three hours). 

### 3.6. Bread Properties

Bran-enriched bread resulted in a darker (increase in 100-L*) and redder (higher a*) crumb and crust than CTRL bread (Table 5). As expected, this phenomenon was more intense in BSW-enriched bread. When BSW was added instead of BUW, also the color crumb (Table 5) showed a significant increase in browning (100-L*) and in redness (a*) levels.

As expected, the addition of bran had a slight negative effect on specific volume, however no significant differences were found between CTRL and BSW. 

**Figure 7:**
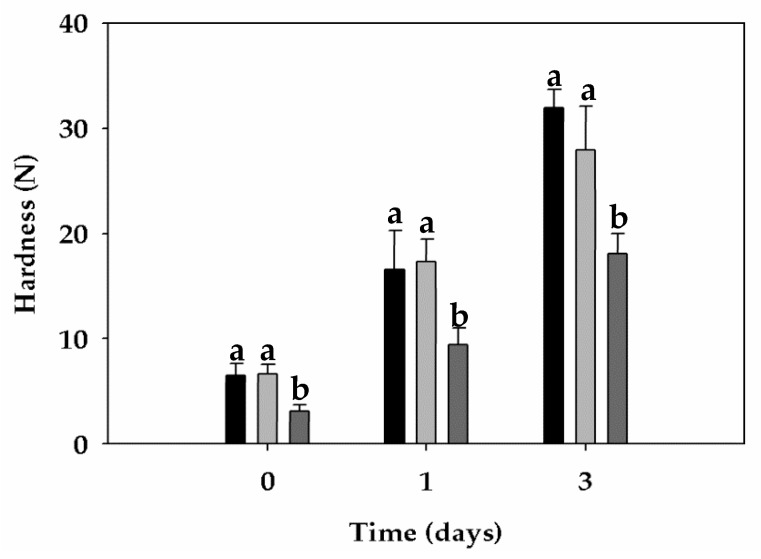
Crumb firmness properties of loaves obtained from refined wheat flour (black), with bran from un-sprouted wheat (light grey), or bran from sprouted wheat (dark grey) during storage. Values associated with different letters (a, b) in the same day are significantly different (one-way ANOVA, LSD test, *p* ≤ 0.05).

The addition of BSW to wheat resulted in a relevant decrease in crumb hardness (3.1 N) compared to the other samples (6.0 N). During storage (up to 3 days), only BSW-samples exhibited higher softness than the CTRL (Figure 7). In addition, the softness of BSW-enriched bread after 3 days was similar in softness to the CTRL and BUW-enriched breads after one day.

To evaluate the kinetic attitude of starch during the retrogradation phenomena, the Avrami equation was applied, fixing the value of Avrami exponent (*n* = 1) as proposed by Michniewicz et al. [29]. The estimated Avrami coefficient *k* for bran-enriched bread samples was higher than that for the CTRL sample. The high coefficient of starch retrogradation with BUW described a fast firming rate. The BSW led to a decrease in the firming rate (Table 5) compared to the BUW and CTRL. Regarding the firmness at infinite time (*A*_∞_) the highest value was found for the CTRL sample, instead the lowest *A*_∞_ was observed in the presence of BSW. The intensity of staling evaluated as the total firmness increment (*A*_∞_–*A*_0_) decreased with the addition of bran, due to the high water binding capacity of fiber. For this parameter, no difference was observed between bran-enriched breads.

## 4. Discussion

Due to the recent interest in using refined flour from sprouted wheat in bread-making as flour improver or ingredient [12,13], it is worthy of interest to investigate the potential use of bran obtained from the milling of sprouted wheat. In this study, the effect of sprouting was assessed on both physical and chemical properties of wheat bran. Moreover, the effect of wheat replacement (at 20% level which is roughly the percentage of bran in wholegrain) with bran from sprouted wheat was studied on both dough rheology and bread-making performance. 

Despite the well-known nutritional benefits of bran, the presence of antinutritional factors (i.e., phytic acid) inhibit the absorption of minerals and vitamins [30]. Several authors have been reported the positive effects of sprouting process on the degradation of phytates in wheat and other cereals [25]. As reported in Table 1, the sprouting conditions applied in the present study caused a significant decrease in phytic acid.

From the technological standpoint, it is well known that the incorporation of fiber into flour negatively affects the textural and sensory properties of cereal-based products. Indeed, numerous negative effects on dough and breads properties have been as attributed to bran, including increased dough stickiness, decreased mixing, fermentation tolerance, volume, and crumb softness [4]. For this reason, it is indispensable to modify the structural properties of fiber (i.e., by increasing the soluble fraction) to improve the quality of bran-enriched foods. Specifically, the soluble fraction of the dietary fiber contributes to the creation of the viscosity of the liquid phase of the system [31]. The sprouting effects on fiber content are reported to be varied and unclear, with increased, decreased and no change reported for several grains [32]. The sprouting process carried out under the conditions applied in this study determined a slight decrease in the TDF and IDF content, instead no changes was observed in terms of SDF (Table 1). A similar trend in dietary fiber change was also found by Koehler et al. [33] up to 4 days of sprouting at about 20 °C. The decrease in TDF is likely due to the breakdown of the water extractable dietary fiber components to smaller molecules which are not precipitable in ethanol, thus not counted as dietary fiber by the method used [8,34]. On the other hand, the decrease in IDF might be due to the xylanase activity developed during sprouting (Table 1). Indeed, such enzymes is the main responsible for the hydrolysis of xylans, the principal component of hemicellulose (about 30% of cell walls) [35]. The changes in dietary fiber component ratio might affect the functional properties of foods, which are mainly related to fiber-water interactions (i.e., water holding capacity (WHC) and water binding capacity (WBC)). The WHC indicates the amount of water that fibers can absorb in the absence of an external force, instead, WBC represents the amount of water that remains bound to fiber after the application of an external force [36]. Sprouting process affected fiber-water interactions as result of the changes induced by the xylanase activity developed during the process (Table 1). Specifically, the decrease in WHC in BSW could be due to the decrease in IDF [37] and perhaps to the lower (even if not statistically significant) content of water-unsoluble arabinoxylans, compared to BUW (Table 1). Indeed, water-unsoluble arabinoxylans are characterized by a stronger WHC compared to the water-soluble ones [38]. The significant decrease in IDF and the slightly increase in water-soluble arabinoxylans in BSW could also explain its lower ability to absorb water in dough system (see farinograph index; Table 2) compared to BUW. Indeed, these two components are responsible for the WHC of the fiber-enrich foods [39]. The xylan-degradation might profoundly modify the bran attitude in bread-making, likely due to modification in water distribution caused by losing their strong water-holding capacity [40]. In order to improve the bran enriched-bread acceptance, generally hemicelluloses (e.g., endoxylanase) are used in bread-making [39], since the soluble arabinoxylans released contribute to forming a hydrated network, together with gluten-forming proteins [41].

To understand the effects of sprouting on fiber-protein interactions, the bran-enriched flours were assessed by means of GlutoPeak test. Generally, flour with good bread-making quality is characterized by a much slower buildup in dough consistency, require more time to reach peak consistency and show high maximum torque [26]. Regardless the type of bran, the profile of bran-enriched flour samples suggested a weakening of gluten network, since the presence of fiber causes deleterious effects on dough structure due to the dilution of the gluten matrix [3]. Several authors postulated that the worsening of dough attributes cannot be related to gluten dilution only [4], but also to physical, chemical, and/or biochemical bran properties [5]. As reported in Table 2, BSW-blend profile showed longer peak maximum time than BUW-blend, suggesting that sprouting attenuated the negative impact on gluten aggregation properties. A possible reason is that the higher insoluble content of dietary fiber in the BUW-blend allowed the formation of a viscous film that hindered the interaction among flour particles [42], preventing the formation of a strong gluten network like that of BSW-blend. In addition, even the decrease in phytic acid content in the BSW-sample could play a role in determining a less negative impact on gluten aggregation. Indeed, phytic acid might react with gluten-forming proteins worsening their aggregation capacity [43,44]. Last but not least, the amount of ferulic acid—whose interaction with proteins negatively impact the gluten formation [5]—decreased upon sprouting [45]. 

The gluten weakening resulted from the addition of bran was also observed in dough system, evaluated as mixing properties (i.e., dough development time and stability, and degree of softening) (Table 2). In this system, the addition of BSW had a greater impact than BUW (Table 2). This result might be due to proteases expressed in the bran layers during sprouting process, as shown by CLSM (Figure 1D). The effect of proteases is evident during the farinograph test that provides for long mixing time (about 20 min). In accordance, Marti et al. [12] reported that after 48 h of sprouting, proteases in refined flour increased from 0.66 to 1.43 unit*g^−1^. 

Despite the worsening of mixing properties, the enzymatic activity developed during sprouting did not further worsen the extensibility properties of the dough (Table 3). Indeed, differences between BUW- and BSW-blends were observed only after 135 min in terms of resistance to extension and energy (Table 3). The high capacity of the BSW-blend to maintain the extensibility properties could be attributed to the new interactions established between the gluten network and the fiber able to partially contrast the negative effect of the hydrolytic activity developed during the sprouting process and activated during the test (Table 1). Selinheimo et al. [46] reported that proteases—specifically laccase which could play some role in the pre-harvest sprouting—increase the maximum resistance and, at the same time, decrease the extensibility of the dough. Moreover, it was reported that the use of cell-wall degrading enzyme (xylanases) increases the resistance to extension in wheat doughs [46,47]. The parameter of resistance to extension is an indicator of dough-handling properties and it is positively correlated to dough volume, since doughs characterized by a high resistance to deformation show better performance in bread-making [48]. In that respect, the more resistant gluten of BSW-blend led to a bread characterized by higher volume than BUW, although not statistically significant (Table 5). We expect that differences between the samples will be highlighted whenever the bread-making trials will be carried out on larger scale (i.e., >250 g loaf), instead of micro-scale, as in this study, or using different leavening processes.

The improvement of bread in the presence of BSW (Table 5) can also be linked to the xylanase activity developed during the sprouting process (Table 1). Indeed, in bread-making, xylanases are commonly used to improve dough handling properties [49], and loaf volume [49,50]. 

As regards crumb firming rate, it was evaluated by using the Avrami equation (1). In general, samples with high firmness rate constant (k) values are characterized by fast crumb firming kinetics, whereas, low *k* values lead to a slow firming kinetics. Both firmness rate constant and initial crumb firmness showed a decrease when BSW was used (see crumb firmness kinetic values; Table 5) instead of BUW. This result might indicate that the xylanase activity developed during sprouting process improved the initial crumb texture and its firming rate of the resulting bread (Table 5). Indeed, the positive effects of xylanases on bread are related to the cleavage of the backbone of arabinoxylans, with the consequent release of water and decrease in water-insoluble pentosane [51]. In addition to xylanases, also α-amylases have positive effects on dough development and crumb staling. The greater presence of fermentable sugars in the BSW (Table 1)—consumable by yeasts for their growth and CO_2_ production—might have contributed positively to the decrease in leavening time (Table 4) and to the greater volume of the corresponding bread (Table 5) [52]. The presence of BSW enhanced the textural properties of loaves, since the crumb hardness—already after two hours of baking—was significantly lower compared to the other samples. This difference is not due to either the crumb moisture, which was not statistically different among the samples (data not shown), or to the crumb porosity, as the BSW-bread was characterized by the lowest value of this index. On the contrary, the crumb texture improvement is due to the α-amylase activity, whose effects have been demonstrated in several studies [53,54,55]. Furthermore, the high α-amylase activity of the BSW had positive effects on firming rate of the corresponding bread crumb (Table 5). 

In conclusion, this research provides information about the effects induced by sprouting process on the chemical and physical properties of bran and how these features affect its interaction with water and gluten in both slurry (using the GlutoPeak test) and dough systems. Last but not least, the effect on bread were considered. Unlike the numerous studies present in the literature, where the enzymatic treatments were conducted directly on bran, isolated after milling (i.e., fermentation, addition of enzymes), in this study the bio-technological treatment was performed directly on the wheat kernels. Using bran from sprouted wheat in bread-making has led to positive effects in terms of gluten aggregation kinetic, bread volume and crumb softness, compared to the use of conventional bran. Thus, bran from sprouted wheat might represent a valid strategy to produce staple foods rich in fiber with high quality traits. Moreover, the use of milling by-products is a good approach to decrease food losses. Finally, results from this study might encourage the use of bran from sprouted wheat in small amount in bread formulations to replace the use of exogenous enzymatic improvers and create clean-label products.

## Figures and Tables

**Table 1:** Effect of sprouting on wheat bran characteristics.

	Bran from Unsprouted Wheat (BUW)	Bran from Sprouted Wheat (BSW)
Total starch	18.3 ± 0.5	26.9 ± 2.0 **
Arabinoxylans		
Total	14.4 ± 0.5	12.0 ± 0.7
Soluble	0.22 ± 0.01	0.29 ± 0.08
Phytic acid	13.98 ± 0.46	11.29 ± 0.02 *
Total Sugar	3.0 ± 0.9	7.0 ± 2.2 *
Glucose	0.14 ± 0.01	0.53 ± 0.02 *
Fructose	0.05 ± 0.01	0.20 ± 0.00 *
Sucrose	2.02 ± 0.20	4.99 ± 0.00 *
Raffinose	0.79 ± 0.02	n.d.
Maltose	n.d.	1.29 ± 0.03
Total Dietary Fiber (TDF)	45.4 ± 0.6	40.2 ± 0.9 *
Soluble (SDF)	2.2 ± 0.4	2.0 ± 0.3
Insoluble (IDF)	43.1 ± 0.2	38.2 ± 0.6 *
α-amylase activity	0.094 ± 0.003	143 ± 16 *
Xylanase activity	0.16 ± 0.04	0.35 ± 0.03 *
Water Holding Capacity	4.9 ± 0.1	4.5 ± 0.1 *
Water Binding Capacity	3.9 ± 0.2	3.7 ± 0.2

Values associated with asterisks in the same row are significantly different (*t*-test, * *p* ≤ 0.05; ** *p* ≤ 0.001); n.d.: not detectable. Compositional and hydration property data are expressed as g/100g sample (d.b.). α-amylase activity and xylanase activity are expressed as Ceralpha Units (CU)/g flour and as activity/g flour, respectively. TDF, total dietary fiber; SDF, soluble dietary fiber; IDF, insoluble dietary fiber.

**Table 2:** Gluten aggregation and mixing properties of refined wheat flour alone, with bran from un-sprouted wheat, or bran from sprouted wheat.

			Refined Wheat Flour (CTRL)	CTRL + BUW	CTRL + BSW
GLUTEN AGGREGATION PROPERTIES (GlutoPeak Test)	Peak maximum time	(s)	192 ± 3 ^c^	65 ± 1 ^a^	93 ± 4 ^b^
	Maximum torque	(BE)	40.3 ± 0.6 ^a^	45.7 ± 0.6 ^c^	43.7 ± 2 ^b^
	Total Energy	(GPE)	3567 ± 112 ^c^	1914 ± 25 ^a^	2059 ± 35 ^b^
MIXING PROPERTIES (Farinograph Test)	Water absorption	(%)	58.1 ± 0.2 ^a^	65.6 ± 0.5 ^c^	61.8 ± 0.5 ^b^
	Development time	(min)	10.8 ± 0.3 ^c^	5.6 ± 0.3 ^b^	4.5 ± 0.5 ^a^
	Stability	(min)	23 ± 2 ^c^	12 ± 2 ^b^	6.4 ± 0.07 ^a^
	Degree of softening	(FU)	15 ± 1 ^a^	44 ± 5 ^b^	103.5 ± 0.7 ^c^

At different letters (a, b, c) in the same row, correspond significant differences (one-way ANOVA, LSD test, *p* ≤ 0.05). BUW: bran from unsprouted wheat; BSW: bran from sprouted wheat. Peak maximum time: indicates the time to obtain the Maximum Torque; Maximum torque: indicates the peak following the aggregation of the gluten proteins; Energy: indicates the area under the curve from the beginning of the analysis up to 15 s after the Maximum Torque. BE: Brabender Equivalent; GPE: GlutoPeak Equivalent. Water absorption: amount of water to obtain a dough with optimal consistency (500 ± 20 FU); Dough development time: time required for the dough development; Stability: point between arrival time (when the top of the curve reaches the 500 FU line) and departure time (when the top of the curve leaves the 500 FU line); Degree of softening: difference between the 500 FU line and the dough consistency at 12 min after the dough development. FU: Farinograph Units.

**Table 3:** Extensibility properties of refined wheat flour alone, with bran from un-sprouted wheat, or bran from sprouted wheat.

	Resting Time	CTRL	CTRL + BUW	CTRL + BSW
Extensibility (E) (mm)	45 min	209 ± 11 ^b^	119 ± 6 ^a^	132 ± 4 ^a^
Resistance to extension (R) (BU)		353 ± 46 ^a^	423 ± 29 ^a^	431 ± 1 ^a^
R/E ratio		1.7 ± 0.3 ^a^	4.0 ± 0.4 ^b^	3.3 ± 0.1 ^b^
Energy (cm^2^)		155 ± 9 ^b^	76 ± 2 ^a^	88 ± 2 ^a^
Extensibility (E) (mm)	90 min	182 ± 7 ^b^	104 ± 6 ^a^	109 ± 3 ^a^
Resistance to extension (R) (BU)		405 ± 63 ^a^	512 ± 26 ^a,b^	583 ± 41 ^b^
R/E ratio		2.2 ± 0.3 ^a^	4.9 ± 0.5 ^b^	5.4 ± 0.2 ^b^
Energy (cm^2^)		147 ± 31 ^b^	77 ± 4^a^	94 ± 11 ^a,b^
Extensibility (E) (mm)	135 min	185 ± 2 ^b^	97 ± 2 ^a^	97 ± 1 ^a^
Resistance to extension (R) (BU)		430 ± 45^a^	609 ± 32 ^b^	721 ± 7 ^c^
R/E ratio		2.3 ± 0.3 ^a^	6.0 ± 0.3 ^b^	7.5 ± 0.1 ^c^
Energy (cm^2^)		163 ± 4 ^b^	85 ± 5 ^a^	101 ± 3 ^a^

At different letters (a, b, c) in the same row, correspond significant differences (one-way ANOVA, LSD test, *p* ≤ 0.05). CTRL: refined wheat flour; BUW: bran from unsprouted wheat; BSW: bran from sprouted wheat. E: dough extensibility; R: resistance to constant deformation after 50 mm stretching; R/E: ratio of resistance to extension and extensibility.

**Table 4:** Leavening properties of refined wheat flour alone, with bran from un-sprouted wheat, or bran from sprouted wheat.

	CTRL	CTRL + BUW	CTRL + BSW
Maximum dough height (mm)	51 ± 3 ^b^	47 ± 1 ^a^	47 ± 1 ^a^
Time of maximum dough development (min)	180 ± 0 ^c^	161 ± 2 ^b^	131 ± 6 ^a^
Dough height at 180 min (mm)	51 ± 3 ^b^	44 ± 1 ^a^	39 ± 1 ^a^
Weakening coefficient at 180 min (%)	n.d.	6 ± 1 ^b^	17.5 ± 0.4 ^a^
Total CO_2_ (mL)	1129 ± 52 ^a^	1301 ± 42 ^a,b^	1476 ± 84 ^b^
Retained CO_2_ (mL)	1064 ± 45 ^a^	1197 ± 29 ^b^	1271 ± 46 ^b^
Released CO_2_ (mL)	65 ± 7 ^a^	104 ± 12 ^a^	205 ± 38 ^b^
CO_2_ retention coefficient (%)	94 ± 1 ^b^	92 ± 0.1 ^b^	86 ± 2 ^a^
Porosity time (min)	n.d.	103 ± 4 ^b^	76 ± 4 ^a^

At different letters (a, b, c) in the same row, correspond significant differences (one-way ANOVA, LSD test, *p* ≤ 0.05). n.d.: not detectable. CTRL: refined wheat flour; BUW: bran from unsprouted wheat; BSW: bran from sprouted wheat. Maximum dough height: maximum height reached during the leavening; final dough height: height reached by the dough at the end of the analysis; maximum height: maximum height relative to gas production; porosity time: time when the dough porosity appears; total CO_2_: total amount of CO_2_ produced; CO_2_ retained: amount of CO_2_ retained in the dough; CO_2_ released: amount of CO_2_ released from the dough; CO_2_ retention coefficient: ratio between CO_2_ retained and total CO_2_.

**Table 5:** Properties of bread from refined wheat flour alone, with bran from un-sprouted wheat, or bran from sprouted wheat.

		CTRL	CTRL + BUW	CTRL + BSW
Bread	Specific volume (mL/g)	2.7 ± 0.6 ^b^	2.2 ± 0.1 ^a^	2.4 ± 0.1 ^a,b^
Crust	Browning (100-L*)	53 ± 4 ^a^	60 ± 4 ^b^	60 ± 1 ^b^
	Redness (a*)	9 ± 3 ^a^	12 ± 1 ^b^	12 ± 1 ^b^
	Yellowness (b*)	25 ± 2 ^b^	20 ± 5 ^a^	19 ± 1 ^a^
Crumb	Browning (100-L*)	48 ± 3 ^a^	52 ± 2 ^b^	57 ± 2 ^c^
	Redness (a*)	−1.3 ± 0.2 ^a^	3.80 ± 0.3 ^c^	3.2 ± 0.4 ^b^
	Yellowness (b*)	18 ± 1 ^b^	16 ± 1 ^a^	16 ± 1 ^a^
Crumb firming kinetics	*A*_0_ (N)	6.48	6.62	3.12
	*A*_∞_ (N)	66.01	34.55	31.35
	*A*_∞_-*A*_0_ (N)	59.53	27.93	28.23
	*k* (h^-n^)	0.186	0.480	0.252

At different letters (a, b, c) in the same row, correspond significant differences (one-way ANOVA, LSD test, *p* ≤ 0.05). CTRL: refined wheat flour; BUW: bran from unsprouted wheat; BSW: bran from sprouted wheat. *A*_0_: hardness at times zero; *A*_∞_: hardness at infinity; *k*: rate constant; n: Avrami exponent.
